# Bacteria in the cavity-restoration interface after varying periods of clinical service — SEM description of distribution and 16S rRNA gene sequence identification of isolates

**DOI:** 10.1007/s00784-022-04473-2

**Published:** 2022-04-01

**Authors:** Roopinder Kaur Arora, Nicola J. Mordan, David A. Spratt, Yuan Ling Ng, Kishor Gulabivala

**Affiliations:** 1 Unit of Endodontology, Departments of Restorative Dentistry, Microbial Diseases, UCL Eastman Dental Institute, University College London, Bloomsbury Campus, Rockefeller Building, 21 University Street, London, WC1E 6DE UK; 2 Biomaterials and Tissue Engineering, UCL Eastman Dental Institute, University College London, London, UK

**Keywords:** Bacteria, Microleakage, Cavity-amalgam interface, SEM, Bacterial identification

## Abstract

**Objectives:**

To use extracted human teeth with amalgam (*n* = 26) or GIC (*n* = 3) restorations in service up to 20 years to evaluate microbiota at the cavity/restoration interface by SEM or culture.

**Materials and methods:**

Extracted teeth with intracoronal restorations (*n* = 20) of known history (2–20 years) were fixed, split, and prepared for SEM to ascertain the pattern and structure of bacterial aggregates on cavity and restoration surfaces. Another 9 teeth were anaerobically decontaminated, split and sampled (cavity/restorations), and cultured (anaerobically, aerobically); recovered isolates were identified by 16S rRNA gene sequencing.

**Results:**

SEM showed rods, cocci, and filaments in 11/20 teeth (55%) on cavity and corresponding restoration surfaces; 4/20 (20%) on neither surface; 1/20 (5%) on just cavity; and 4/20 (20%) on just restoration. Microbial growth extended from marginal openings into the deeper interfacial microspace to varying extents but was not always evident. Restoration size or age did not predict bacterial presence. Bacteria-free surfaces (cavity/amalgam) showed possible calcification. Cultivation yielded 160 isolates, mainly Gram-positive (86%) and facultative (81%); and morphotypes of rods (43%), cocci (36%), and cocco-bacilli (18%) belonging to *Actinobacteria* (45%) and *Firmicutes* (50%). The most frequent genera were *Staphylococcus*, *Streptococcus*, *Actinomyces*, and *Lactobacillus.* Biofilms on cavity and restoration appeared independent of each other.

**Conclusions:**

Cavity and amalgam surfaces were independently colonised and some not. The penetration of microbiota into marginal gaps varied; resembled root caries and was dominated by Gram-positive species.

**Clinical relevance:**

Marginal gaps around restorations are unavoidable but are not always colonised by bacteria after long-term clinical service. Calcification of biofilms in the restorative interface may prevent further colonisation. The viable microbiota in the restorative interface resembled root caries and may be subject to ecological fluxes of activity and arrest and therefore preventative management.

## Introduction

Dentine exposure through restorative treatment causes immediate but potentially reversible pulp inflammation; however, sustained, accumulative injury across the pre-, intra- and post-operative phases may help propagate the inflammation to the point of the pulp’s ultimate demise [1–3]. Irreversible pulpal damage has been historically attributed to operative [4], chemical [5], or microbial [6–9] factors. The current view is that a complex inter-relationship between the reactive capacity of the pulp, remaining dentine thickness, nature of the restorative material and the nature and proximity of its adaptation to the cavity, influences microbial colonisation (leakage) [3, 10, 11].

Regardless of the material used, the restorative imperative is to achieve the best adaptation of the filling material to the cavity wall to exclude microleakage, which is the penetration of oral fluids and microorganisms along the restorative material/cavity wall interface and its critical colonisation. Even modern adhesive dental materials do not guarantee permanent, durable adaptation to the cavity wall in the oral environment [12–15]. There is always a microscopic gap at the interface, clinically undetectable by the finest tipped (40 μm) dental probe, allowing penetration of fluids and bacteria from the oral cavity [11, 16]. Such leakage is associated with a variety of clinical consequences, including post-restoration dentine sensitivity, marginal staining (tooth or restorative material), restorative material degradation, dentine softening, frank secondary caries, pulpal and periapical inflammation, and failure of root canal treatment [17, 18]. Although differences may be apparent in the clinical frequency of such problems between various restorative materials [11, 18], the probability and predictability of the depth of microbial colonisation into the marginal interface lacks clarity [19].

Matharu et al. [20] studied short-term (8 weeks) microbial leakage dynamics using a constant-depth-film-fermentor (CDFF) model with standardised amalgam–restored cavities in bovine dentine. The study refined a method of splitting the dentine/restoration complex to expose their interface for direct microscopic examination and to enable sampling of bacteria for culture.

The aim of this study was to use an ex vivo sample of teeth with plastic (mainly amalgam) restorations in functional clinical service for various periods in the human oral environment (and judged to be “serviceable”), to evaluate the microbiota at the cavity/restoration interface, by (1) scanning electron microscope (SEM) description of biofilm presence and topography and (2) comparative 16S rRNA gene sequence identification of cultivable isolates.

## Materials and methods

### Selection and storage of sample teeth

#### SEM study

Approval was granted for the use of extracted teeth from the UCL Eastman Biobank (study number: 1301) and included informed consent in writing for donation of their extracted teeth, which were stored as individual specimens in unique and individual containers for the study. The Eastman Biobank is approved by the Yorkshire & Humber—Leeds East Research Ethics Committee (REC) to use samples or surplus tissues (including extracted teeth) donated by patients for research purpose (REC reference number: 12/YH/0111). Twenty-seven permanent teeth with serviceable intra-coronal restorations (occlusal, occluso-proximal, mesio-occluso-distal, buccal) and no previous pulp therapy were obtained (by two participating general dental practitioners [GDP] in separate general dental practices) with informed consent from patients undergoing routine extractions for general restorative or periodontal reasons. The teeth were immediately placed in specimen tubes containing 3% glutaraldehyde (Agar Scientific, Stansted, UK) in 0.1 M sodium cacodylate buffer (Agar Scientific, Stansted, UK) for fixation. The responsible GDP provided information on tooth designation, its restoration history, restoration site, number of surfaces, presence of cavity lining, restoration-placement date, periodontal involvement, radiographic findings, and reason for extraction. Inclusion required the requisite information, as well as absence of visible cracks or fractures of the tooth. The sample characteristics are summarised in Table 1.

**Table 1:** Clinical history of sample teeth and restorations for SEM study

Tooth	Restoration site	Restorative material	Liner/base	Years restoration present	Reason for extraction	Radiographic findings	Periodontal involvement
1	O	Amalgam	Ca(OH)_2_	4 years	Periodontal	Normal PDL space	Yes
2	MOD	Amalgam	Ca(OH)_2_	2 years	Periodontal	Widened PDL	Yes
3	O	Amalgam	Ca(OH)_2_	13 years	Periodontal	Normal PDL space	Yes
4	MOD	Amalgam	Ca(OH)_2_	12 years	Mesial caries, distal intact	Widened PDL	No
5	MO	Amalgam	Ca(OH)_2_	11 years	Pain	Normal PDL space	No
6	O	Amalgam	Ca(OH)_2_	20 years	Mesial caries, distal intact	Periapical radiolucency	No
7	B	GIC	None	Not known	Mobile	Widened PDL	Yes
8	MOD	GIC	None	4 years	Causing pain, non-vital	Periapical radiolucency	No
9	MOD	Amalgam	None	5 years	Periapical abscess	Periapical radiolucency	Yes
10	MO	Amalgam	Ca(OH)_2_	5 years	Periodontal abscess	Widened PDL	Yes
11	B	GIC	None	11 years	Mobile	Periapical radiolucency	Yes
12	O	Amalgam	Ca(OH)_2_	20 years	Mobile	Widened PDL	Yes
13	O	Amalgam	Ca(OH)_2_	9 years	Mobile & pain	Widened PDL	Yes
14	O	Amalgam	Ca(OH)_2_	Not known	Distal caries, mesial intact	Widened PDL	No
15	O	Amalgam	Ca(OH)_2_	11 years	Mobile	Widened PDL	Yes
16	MOD	Amalgam	None	15 years	Mobile	Normal PDL space	Yes
17	MO	Amalgam	Ca(OH)_2_	12 years	Pain	Periapical radiolucency	No
18	O	Amalgam	Ca(OH)_2_	15 years	Mobile	Widened PDL	Yes
19	DO	Amalgam	Ca(OH)_2_	Not known	Mobile	Widened PDL	Yes
20	O	Amalgam	Ca(OH)_2_	10 years	Mobile	Widened PDL	Yes

*B* Buccal, *MO* mesio-occlusal, *D* distal, *DO* disto-occlusal, *O* occlusal, *MOD* mesio-occlusal-distal, *PDL* periodontal ligament space

#### Cultivation study

In addition, nine freshly extracted permanent teeth with amalgam restorations, no obvious cracks, and no previous pulp therapy were obtained from patients attending the Oral Surgery Department at the Eastman Dental Hospital (UCLH Trust) with informed consent as described above. If a carious lesion was present, it was ensured that at least one aspect of the cavity wall/restoration interface was intact, and it was this that was studied. The same questionnaire was used, and the sample characteristics are summarised in Table 2.

**Table 2:** Clinical history of teeth and restorations for cultivation study

Tooth	Alpha-numeric notation	Restoration site	Restorative material	Liner/base	Years restoration present for	Reason for extraction	Radiographic findings	Periodontal involvement
1	LL8	O	Amalgam	None	2 years	Mesial caries, distal intact	Periapical radiolucency	Yes
2	LL6	MOD	Amalgam	None	5 years	Mesial caries, distal intact	Widened PDL	No
3	LL8	O	Amalgam	None	20 years	Cheek biting	Normal PDL space	No
4	UR7	MO	Amalgam	Ca(OH)_2_	4 years	Mesial caries, distal intact	Periapical radiolucency	Yes
5	UL8	O	Amalgam	None	2 years	Distal caries, mesial intact	Normal PDL space	No
6	UL8	O	Amalgam	None	4 years	Distal caries, mesial intact	Widened PDL	No
7	UR5	O	Amalgam	None	14 years	Perio	Lateral periradicular radiolucency continuous with apical lesion	Yes
8	UL8	MOD	Amalgam	Ca(OH)_2_	8 months	Severe pain history	Normal PDL space	No
9	UR8	MO	Amalgam	None	7 years	Pain	Normal PDL space	No

*O* Occlusal, *MO* mesio-occlusal, *MOD* mesio-occlusal-distal, *PDL* periodontal ligament

The nine teeth obtained for the cultivation study were placed immediately into sterile vials, transported to the microbiology laboratory within 1 h of extraction, and placed in the anaerobic chamber with an atmosphere of 80% N_2_, 10% CO_2_, and 10% H_2_ at 37 °C and 100% relative humidity (MACS-MG 1000, Anaerobic workstation Don Whitley Scientific, Skipton, UK).

### SEM analysis

**Fig. 1:**
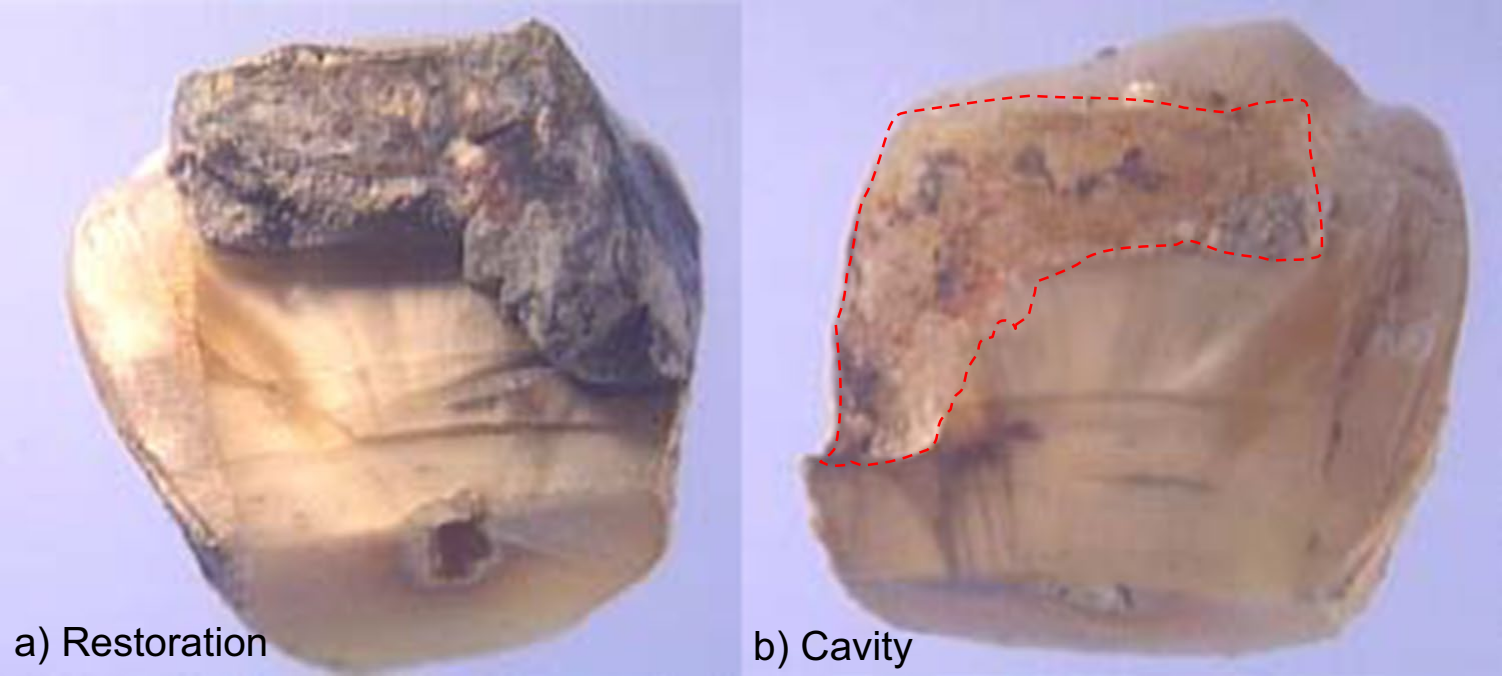
Photograph of a tooth after splitting, with the restoration in one half (mag × 17)

The 27 sample teeth were sectioned in a ductless fume cupboard (Labcaire, Clevedon, UK) with an ultra-fine diamond disc (Super-Diaflex discs, Claudius Ash, Potters Bar, UK) at the cemento-enamel junction. The crown was pre-grooved with an ultra-thin diamond disc and embedded in sterile lab putty (Coltene, Whaledent, Mahwah, NJ, USA) to cushion the crown, whilst being split into two using an osteotome, such that the restoration remained in one half of the split tooth (Fig. 1). Each specimen was then prepared for scanning electron microscopy.

The tooth sections were dehydrated in a graded series of ethanol: 20%, 50%, 70%, and 90% each for 15 min and 100% for 3 × 10-min changes. The samples were transferred into hexamethyldisilazane (HMDS) (TAAB Laboratories Ltd, Reading, UK) for 5 min, then removed and left in a medium-flow fume cupboard for the HMDS to evaporate. Each tooth segment was carefully orientated and mounted onto aluminium stereoscan stubs (Agar Scientific, Stansted, UK) with carbon conductive cement (Neubauer Chemikalien, Munster, Germany). The dried, mounted specimens were sputter-coated with gold/palladium in a Polaron E5000 Sputter Coater (Quorum Technologies Ltd, Newhaven, UK), and viewed in a Cambridge Stereoscan 90B SEM (Cambridge Inst., Cambridge, UK). Digital images were recorded with an I-Scan 2000 (ISS Group, Manchester, UK).

### Bacterial cultivation

Excess tissue was removed from the tooth surface with a sterile scalpel blade, and the tooth was surface decontaminated by swabbing with sterile swabs soaked in 30% (w/v) hydrogen peroxide (Sigma, Poole, UK), followed by iodine solution 10% (w/v) (Seton Healthcare Group, Oldham, UK) for 60 s each. The final solution was neutralised with sterile sodium thiosulphate 5% (w/v) (BDH Laboratory Supplies, Poole, UK) (Ng et al. 2003). The teeth were sectioned within the anaerobic chamber with an ultra-fine diamond disc (Super-Diaflex discs, Claudius Ash, Potters Bar, UK) at the cemento-enamel junction. The crown was pre-grooved, embedded in sterile lab putty (Coltene, Whaledent, Mahwah, NJ, USA), and split into two with an osteotome such that the restoration remained in one half of the split tooth. The crown was again surface decontaminated on the external surface avoiding the internal surface.

Three ISO size 15 sterile paper points (Roeko, Langenau, Germany), pre-soaked in reduced transport fluid (RTF) [21], were used to sample the decontaminated surface and placed into 1 mL of RTF as a control. The corresponding exposed interfacial amalgam and dentine surfaces were successively sampled using increasingly abrasive sampling methods, progressing from three pre-soaked ISO size 15 paper points, to a hand-held round, carbon steel (size 3) bur (Claudius Ash, Potters Bar, UK), and then finally to a similar bur rotating in a handpiece (Georg Schick Dental GmbH, Schemmerhofen, Germany). The purpose was to account for any possible mineralised resident plaque or possible softened dentine. Each of the independent samples was transferred to a vial containing 1 mL of RTF and vortexed for 1 min: yielding a total of 6 samples per tooth and 54 samples in all.

Fifty microlitres of each sample was plated on anaerobe agar (Bioconnections, Leeds, UK) containing 5% horse blood and blood agar (Bioconnections) and incubated in anaerobic or aerobic conditions for 7 and 3 days, respectively. Total colony forming units (CFU) were determined, and each unique colony morphotype was sub-cultured to purity. The purified colonies were characterised by colony morphology, size, shape, colour, texture, Gram-staining, and catalase test.

### DNA extraction from isolates and PCR amplification of the 16S rRNA gene

Template DNA was extracted and prepared by adding a bacterial colony from each purified isolate to 200 μL of sterile water in an Eppendorf tube and placing in a microwave for 1 min. This was immediately placed on ice and used as a template in PCR amplification (UNO II Biometra, Maidstone, UK) using 10 μL of 10 × NH_4_ reaction buffer, 5 μL of 50 mM MgCl_2_ solution, 10 μL of 200 μM deoxynucleoside triphosphates, 1 μL each of 10 pmol/μL of the respective oligonucleotide primer combinations 27f, 1492r, 357f and 1392r, 0.3 μL of 5 U/μL *Taq* DNA polymerase (Bioline) and 50 μL of template DNA in a total reaction volume of 100 μL. Thirty amplification cycles were performed with a denaturing temperature of 95 °C for 1 min, annealing at 52 °C for 1 min and elongation at 72 °C for 1 min.

### Agarose gel electrophoresis and purification of the amplicons

Ten microlitres of each PCR product was electrophoresed (Mighty Slim™ SX250 Power Supply, Hoefer Scientific Instruments, San Francisco, CA, USA) on a 0.8% agarose gel in Tris Acetate EDTA (TAE) buffer and stained with 5 μL of 10 mg/mL ethidium bromide (Gibco BRL, Life Technologies, Gaithersburg, USA). It was viewed under ultra-violet light (Model T2201, Sigma Chemical Co, St Louis, MO, USA) to confirm the presence and size of PCR amplicons and the image recorded with a digital system (UVP ImageStore 5000, Ultra-Violet products Ltd, Cambridge, UK). QIAquick™ spin PCR purification kit (QIAGEN GmbH, Düsseldorf, Germany) was used according to the manufacturer’s instructions to remove unreacted dNTPs, primers, *Taq* polymerase, and other potential inhibitors from the amplicon sample prior to sequencing.

### Sequencing of the amplicons

A ‘ready reaction mix’ was prepared using ABI Prism™ Bigdye™ Terminator Cycle Sequencing solution (PE Applied Biosystems, Foster City, CA, USA). The DNA sequencing reaction was performed with the Uno II Thermocycler (Biometra, Maidstone, UK) using a cycle sequencing programme. Ninety-nine cycles were performed with an initial rapid thermal ramp to 95 °C for 10 s, 50 °C for 5 s, and 60 °C for 4 min.

### Outcome measures

In the SEM study, the entire cavity/restoration margin and cavity was viewed to map bacterial presence and extent on the apposing surfaces or to note its absence. The observations were analysed through descriptive statistics. In the culture study, the purified, sequenced products were analysed with automated gene sequencer (ABI Prism™ 310 Genetic Analyser, PE Applied Biosystems, Foster City, USA) in the form of an electropherogram, which was edited as necessary. The corrected sequence was compared with both the 16S rRNA sequence database of the Ribosomal Database Project [https://rdp.cme.msu.edu/] and the Basic Local Alignment Search Tool (BLAST) at the National centre for Biotechnology Information (NCBI) [http://www.ncbi.nlm.nih.gov] via the Internet. Strains were designated to probable genus or species level using 16S rRNA sequence similarity threshold of ≥ 98% for species, ≥ 91% ≤ 97.9% sequence similarity for genus and ≤ 90.9% for genus-like strains [22].

## Results

### SEM observational study

Twenty teeth were ultimately selected for analysis from a total of the 27 processed (Table 1); 7 were excluded because of imperfect tooth splits for revealing cavity and apposing restoration surfaces. Most teeth were restored with amalgam (17/20) and the rest (3/20) with glass ionomer cement; all were deemed “clinically serviceable” rather than requiring immediate replacement. Nine had occlusal, four proximo-occlusal, five mesio-occluso-distal, and two buccal restorations. Calcium hydroxide was the only cavity liner used and was present under 70% of the restorations. The lifespans of the restorations ranged from 2 to 20 years with missing information for 3 teeth. Most teeth (16/20) examined by SEM had radiographic signs of apical changes with 11/20 demonstrating widened apical periodontal ligament (PDL) space indicative of probable advanced pulpitis, 5/20 having periapical radiolucencies indicative of probable pulp necrosis and root canal infection, and 4 with normal PDL spaces. Fourteen of the twenty teeth were periodontally involved, and six were not.

**Fig. 2:**
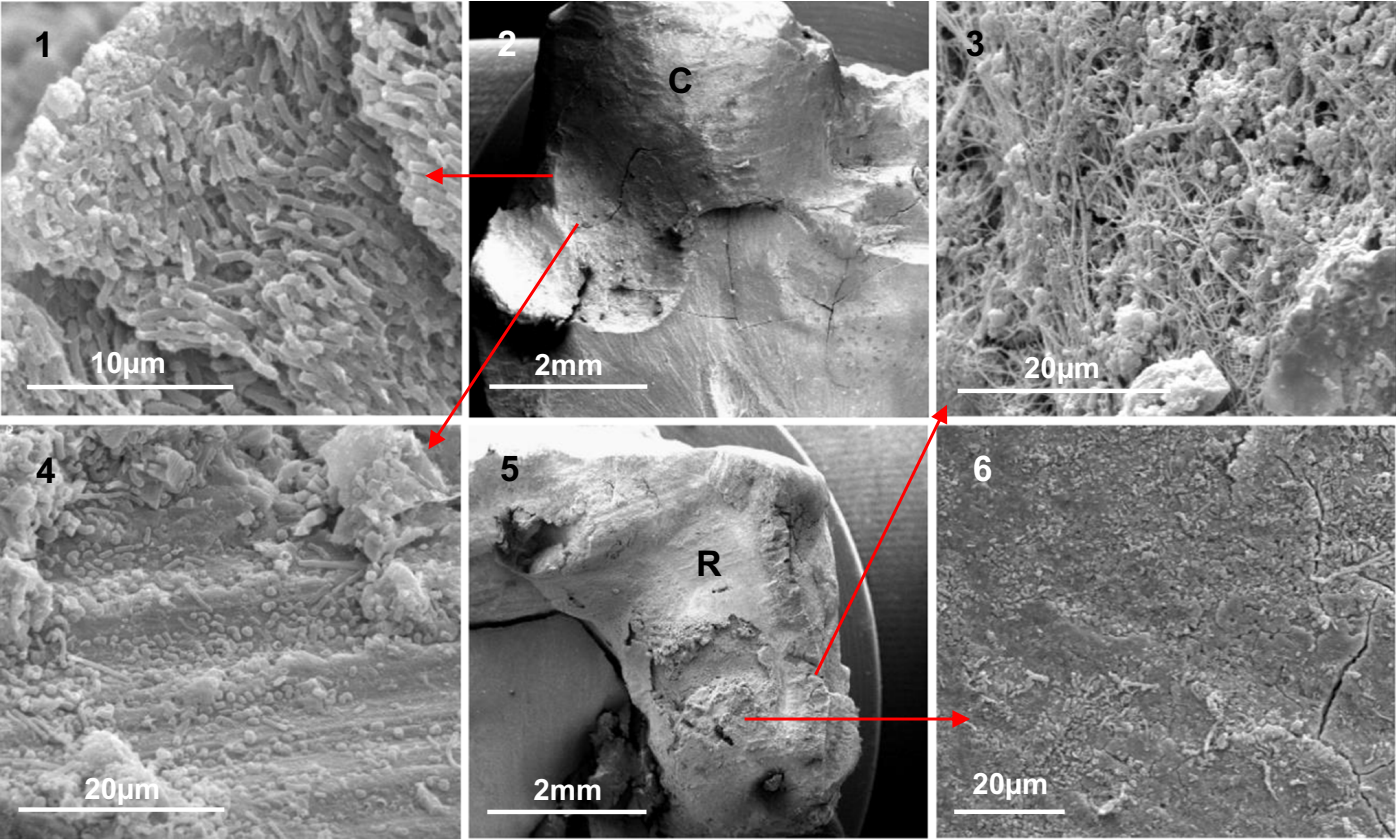
(2) and (5) Low magnification views of an amalgam restoration (R — × 17) and cavity (C — × 15), red arrows indicate higher magnification of specific regions. (1) (× 4090) Plaque at the coronal part of the cavity in (2), whilst further towards the base of the cavity ((4) — × 2040) the bacteria were less abundant, with groups and single cells visible in bur grooves. (3) (× 2120) The plaque, comprising filaments and cocci, at the surface of the amalgam and (6) (× 1080) the biofilm on the amalgam at a deeper level where it comprised mostly cocci and rods

**Fig. 3:**
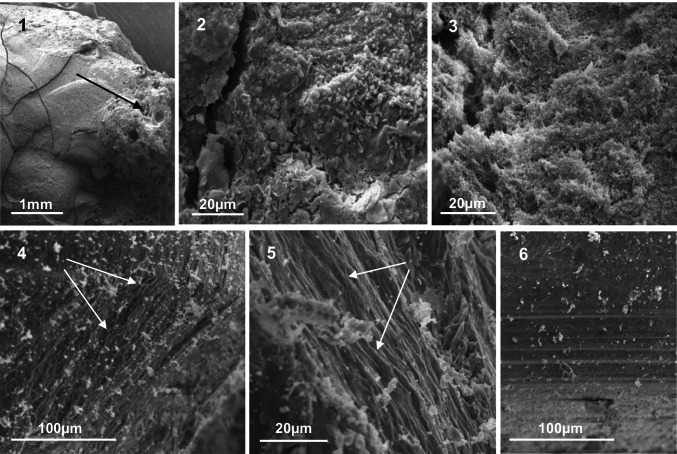
The GIC restorations ((1) — × 15.3) had a more varied surface structure than amalgam with some porous regions (black arrow). The associated biofilms were often thick and varied, both on the restoration ((2) — × 1030) and the cavity wall ((3) — × 1080). (4), (5), and (6) The pattern of down-growth of filamentous bacteria (white arrows) over the amalgam restoration ((4) — × 248) and the cavity wall ((5) — × 1020). This down-growth was absent at the base of the restoration ((6) — × 243)

The observational data by tooth are summarised in Table 3. Of the 20 teeth studied, 11 showed the presence of biofilm and individual bacteria (rods, cocci, filaments) on both the cavity wall and the restoration, sometimes in high numbers (Figs. 2 and 3). There were no visible signs of biofilm or obvious bacterial morphotypes on either the cavity or apposing restoration surfaces in 4 teeth. In the remaining 5 teeth, the apposing surfaces appeared to behave independently with seemingly separate biofilms adherent to each; in 1 tooth, microorganisms were evident on the cavity wall surface only, whilst in 4 samples they were present on the restoration only.

**Table 3:** Summary of observations from SEM

Cavity surface				Restoration surface				
Tooth	Cavity	Quantity of bacteria	Type of growth	Restoration	Restoration type	Quantity of bacteria	Type of growth	Periapical status
**1**	✘	-	-	✘	Amalgam	-	-	NPDL
**2**	✘	-	-	✓	Amalgam	*	C	WPDL
**3**	✘	-	-	✘	Amalgam	-	-	NPDL
**4**	✘	-	-	✘	Amalgam	-	-	WPDL
**5**	✘	-	-	✓	Amalgam	*	C	NPDL
**6**	✓	***	D	✘	Amalgam	-	-	PA
**7**	✓	**	C	✓	GIC	**	C	WPDL
**8**	✓	***	D	✓	Amalgam	**	D	PA
**9**	✓	**	D	✓	GIC	**	D	PA
**10**	✓	***	D	✓	Amalgam	***	D	WPDL
**11**	✓	*	C	✓	GIC	**	C	PA
**12**	✓	*	C	✓	Amalgam	*	C	WPDL
**13**	✓	***	D	✓	Amalgam	***	D	WPDL
**14**	✓	**	D	✓	Amalgam	***	D	WPDL
**15**	✓	*	C	✓	Amalgam	*	C	WPDL
**16**	✘	-	-	✓	Amalgam	*	C	NPDL
**17**	✘	-	-	✓	Amalgam	*	C	PA
**18**	✘	-	-	✘	Amalgam	-	-	WPDL
**19**	✓	*	C	✓	Amalgam	*	C	WPDL
**20**	✓	*	C	✓	Amalgam	*	C	WPDL
**Summary**	✘ = 8		D = 6	✘ = 5			D = 5	
	✓ = 12		C = 6	✓ = 15			C = 10	

✘ No bacterial growth; ✓ bacterial growth; C individual colonies; D biofilm downgrowth; *, **, ***rating of quantity of bacteria; *NPDL*Normal periodontal ligament; *WPDL* widened periodontal ligament; *PA* periapical radiolucency

**Fig. 4:**
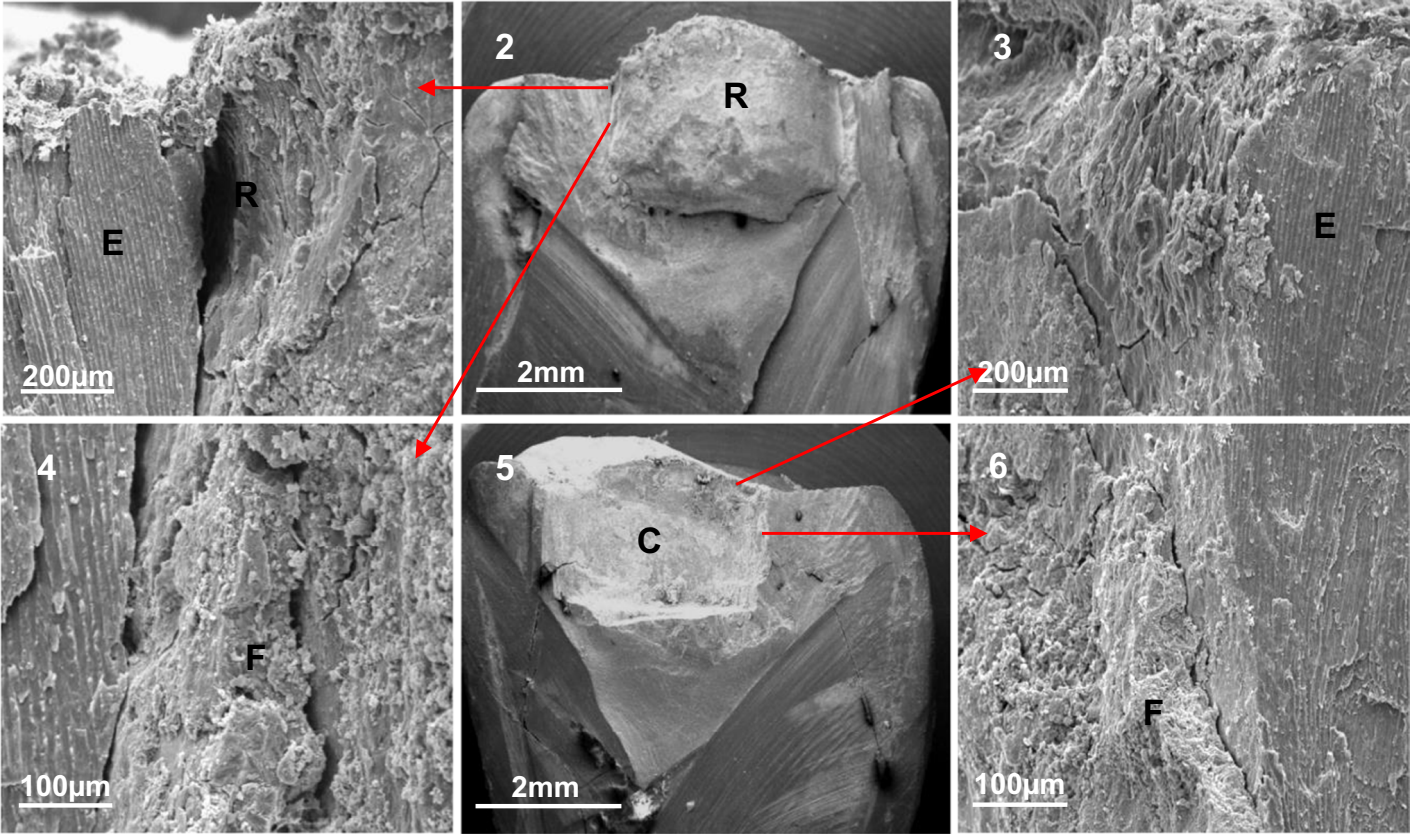
(2) and (5) Low magnification (× 15.3) images of a fractured tooth with the amalgam restoration (R) in (2), where red arrows indicate higher magnification (× 474) images of specific regions. A large gap was present at the restoration (R)/enamel (E) interface ((1) and (2)) with no evident down-growth of overlying plaque. The same area in the cavity (C) (3) was bacteria-free. In (4) and (6), an irregular layer (F) was observed on the restoration and cavity wall, with no bacteria present

A common finding was the presence of an irregular surface with a sometimes ‘lobulated/granular’ appearance in 9 teeth that was different from enamel, dentine, amalgam, and GIC surfaces. This layer was present on both apposing walls of the cavity and restoration in 4 teeth (Fig. 4), on the cavity wall only in a further 4 teeth and on the amalgam surface only in 1 tooth. All these samples had few or no microorganisms, but where the biofilm was thick, the underlying surface was masked. Microorganisms were observed in diminishing numbers towards the deeper parts of the cavity (Fig. 3).

Eight of 17 teeth restored with amalgam had bacteria associated with both the cavity wall and amalgam (Figs. 3 (4), (5), and (6)); whilst one showed microorganisms on the dentine but not the amalgam surface. Of the 17 amalgam restorations, 9 were single-surface occlusal fillings, 6 of which showed bacterial presence on either the tooth or the restoration. The remaining 8 teeth had multiple surface restorations (mesio-occlusal, disto-occlusal, or mesio-occluso-distal) (Table 2); of which, 5 showed bacteria on either the cavity wall or restoration (Table 3). The amalgam restorations were 2–20 years old; 2/4 that were 5 (or fewer) years old showed bacterial presence; and 5/11 that were over 5 years old exhibited microorganisms on either the cavity wall or filling surface (Tables 2 and 3).

All three GIC restorations had bacteria associated with the restoration surface (Figs. 3 (1) and (2)) and cavity wall (Fig. 3 (3)), often as a thick biofilm. The age of one GIC restoration was unknown (Tooth 7), but the remaining two teeth had GIC restorations in place for 4 and 9 years, respectively.

The observed presence of microorganisms on the cavity wall and restoration surfaces in teeth restored with both amalgam and GIC appeared to follow one of two patterns. The first arrangement comprised a mostly continuous bacterial biofilm and was found in 6 of the 16 samples with bacteria, 5 of these having biofilm on both cavity wall and restoration surface. In all samples, these thick biofilms were observed extending from the external oral interface, but only continued to the centre of the cavity in 2 of the 6 samples. In 3 of the 6 samples, the biofilm extended a third of the way into the cavity and in 1 sample extended two-thirds of the way. Filament- and rod-shaped bacteria appeared to predominate in these biofilms.

The second arrangement consisting of discontinuous groups of bacteria (micro-colonies) was found in 10/16 samples. In 6 of these samples (5 amalgam; 1 GIC), bacterial colonies were found on both the cavity and restoration surfaces, but in 4 samples, they were observed only on the amalgam. Rod- and cocci-shaped microorganisms predominated in these small colonies.

### Identification of cultivable isolates from cavity/restoration interface

#### Sample definition and bacterial yield

Of the 9 teeth allocated for cultivation, 6 were wisdom teeth, 2 were molars (first or second), and 1 was a premolar; all had amalgam restorations ranging in age between 8 months to 20 years, and 2 had calcium hydroxide linings. Five teeth exhibited radiographic signs of apical periodontitis, and one was diagnosed as having a perio-endo lesion (Table 2).

**Fig. 5:**
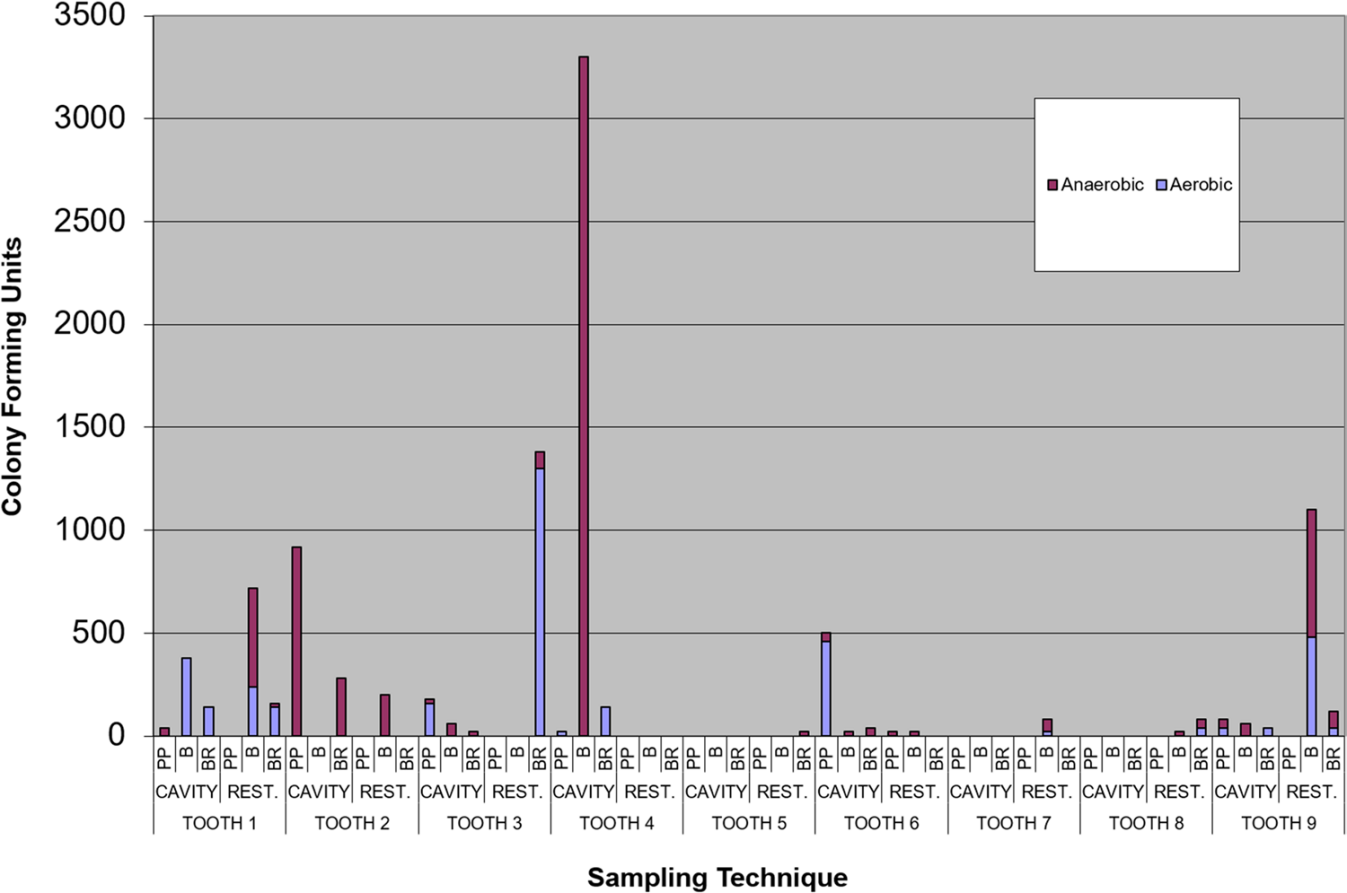
Number of colony-forming units recovered by different sampling techniques. PP = paperpoint; B = hand-held bur; BR = rotating bur

Control samples of the decontaminated tooth surface and putty were culture-negative for all 9 teeth. The hand-held bur gave the highest number of isolates (47%), whilst drilling with the bur (34%) and paper points (19%) yielded relatively smaller numbers (Fig. 5).

All sampled teeth were positive by culture for either the cavity wall or amalgam but not always both (tooth 4, 5, 7, 8). Bacteria were isolated from the cavity wall in 6/9 cases and from the amalgam in 8/9 cases (Table 4). Teeth with occlusal amalgam restorations (*n* = 5) yielded a relatively smaller proportion of isolates (44%) from the cavity/amalgam interface than those (*n* = 4) with two or more surface restorations (MO or MOD) (56%). Approximately 2.7 times more isolates were recovered from the cavity wall and restoration surface when the filling had been present for less than 5 years (73%) compared to when the filling had been present for more than 5 years (27%).

**Table 4:** Main bacterial groups from cavity and restoration surfaces per tooth with sampling designation and periapical status

Tooth	Cavity wall	Restoration surface	Periapical status
1	*Corynebacterium* uncultured *Corynebacterium* sp*.* (PP) *Staphylococcus epidermidis* (B) *Staphylococcus warneri* (BR) *Lactobacillus pontis* (B)	*Lactobacillus zeae* (B) *Staphylococcus epidermis* (B) *Staphylococcus warneri* (BR)	Radiolucency
2	*Actinomyces naeslundii* (PP) *Actinomyces viscosus* (PP) *Actinomyces* sp.*str.* (PP) *Capnocytophaga haemolytica* (PP) *Staphylococcus warneri* (PP) *Streptococcus* sp*.* (PP)	*Actinomyces naeslundii* (B) *Neisseria pharyngis* (PP) 3 strains lost to sub-culture 1 could not be sequenced	Widened PDL
3	*Propionibacterium acnes* (B) *Streptococcus mutans* (PP) *Streptococcus sanguis* (B) 1 strain lost during subculture 1 could not be sequenced	*Actinomyces* sp *(BR)* *Actinomyces viscosus* (BR) *Bacillus thuringiensis* (BR) *Propionibacterium acnes* (BR) *Streptococcus mitis* (BR) *Streptococcus salivarius* (BR)	Normal PDL
4	*Actinomyces* sp. (B) *Actinomyces naeslundii* (B) *Actinomyces viscosus* (B) *Gemella morbillorum* (BR) *Haemophilus parainflunzae* (B) *Lactobacillus zeae* (B) *Neisseria sicca str.* (B *Neisseria flava* (B) *Rothia dentocariosa* (B) *Streptococcus gordonii* (BR) *Streptococcus mitis* (B) *Streptococcus* sp.oral clone (B) *Streptococcus salivarius* (B) *Streptococcus sanguinis* (B) *Veillonella dispar* (B) *Micrococcus luteus* (PP) 2 strains lost to subculture	No isolates	Radiolucency
5	No isolates	*Propionibacterium acnes* (BR)	Normal PDL
6	*Lactobacillus reuteri* (B) *Propionibacterium acnes str.* (BR) *Staphylococcus epidermidis* (BR) *Staphylococcus hominis* (PP) *Staphyloccocus* sp.*str* (PP) *Staphylococcus capitis* (PP)	*Corynebacterium xerosis* (PP) *Lactobacillus* sp*. oral clone* (B) Unable to PCR 2 strains	Widened PDL
7	No isolates	*Paenibacillus polymyxa* (B) *Streptococcus sanguinis* (B) *Staphylococcus caprae* (B)	Lateral radiolucency
8	No isolates	*Paenibacillus polymyxa* (BR) *Staphylococcus* sp*.* (BR)	Normal PDL
9	*Lactobacillus casei* (BR) *Lactobacillus paracasei* (B) *Micrococcus luteus* (PP)	*Streptococcus mutans* (PP) *Actinomyces* sp*.* (B) *Rothia dentocariosa* (B)	Normal PDL

*B* Bur; *BR* Bur in hand-piece; *PP* paper point; *PDL* periodontal ligament space

#### Taxonomic designation of isolates

Overall, cocci accounted for 36% (57/160), bacilli 43% (69/160), cocco-bacilli 18% (29/160), and 3% (5/160) were undecipherable in cellular morphology. Most of the isolates (95%) were Gram-positive, of which the majority were facultative (147/160; 92%) and the rest anaerobic (13/160; 8%).

Of the 160 cultivated isolates, 149 (93%) were given a taxonomic designation by comparative 16S rRNA gene sequencing (Table 5); 7 strains were lost during sub-culture, 2 others were not amenable to PCR amplification, and the PCR amplicons for a further 2 more were not amenable to sequencing. A total of 38 taxa were represented by 4 phyla, 15 genera, and possibly 39 species. Of the 149 strains identified, 50% belonged to the phylum *Firmicutes*, 45% to *Actinobacteria* and the remaining 5% to *Proteobacteria* and *Bacteroidetes*. Only 7% of the strains were identified to species level (98% plus sequence similarity), 56% to genus level (≥ 91% ≤ 97.9% sequence similarity), and 37% were deemed “genus-like” with sequence similarity ranging from 70% ~ 90.0%.

**Table 5:** Bacterial isolates categorized by comparative 16SrRNA sequence at similarities ≥ 98% (species), ≥ 91- 97.9% ≤ (genus) or 90.9% (genus-like)

Phylum designation	Strain identity	No of strains with ≥ 98% sequence similarity (species)	No of strains with ≥ 91–97.9% ≤ sequence similarity (genus)	No of strains with ≤ 90.9% sequence similarity (genus-like)
***Actinobacteria***	***Actinomyces denticolens***	-	-	1
	***Actinomyces naeslundii***	-	17	2
	***Actinomyces viscosus***	-	-	6
	***Actinomyces* sp.**	-	-	6
	***Corynebacterium xerosis***	-	1	-
	*Corynebacterium* uncultured Corynebacterium*	-	-	1
	***Micrococcus luteus***	-	-	2
	***Micrococcus*-like**	2	-	-
	***Propionibacterium acnes***	-	3	3
	***Rothia dentocariosa***	3	17	3
***Sub-total***		**5**	**38**	**24**
***Bacteroidetes***	***Capnocytophaga haemolytica***	-	-	1
***Sub-total***		**0**	**0**	**1**
***Proteobacteria***	***Haemophilus paraphrophilus***	-	-	2
	***Neisseria pharyngis***	-	-	2
	***Neisseria sicca* str**	-	3	-
***Sub-total***		**0**	**3**	**4**
***Firmicutes***	***Bacillus thuringiensis***	1	-	-
	***Gemella morbillorum***	-	2	-
	***Lactobacillus casei***	-	2	-
	***Lactobacillus paracasei***	-	2	-
	***Lactobacillus pontis***	-	2	-
	***Lactobacillus reuteri***	-	-	4
	***Lactobacillus zeae***	-	4	-
	***Paenibacillus polymyxa***	-	-	2
	***Staphylococcus caprae***	-	1	-
	***Staphylococcus capitis***	-	1	2
	***Staphylococcus epidermidis***	-	3	-
	***Staphylococcus haemolyticus***	-	-	3
	***Staphylococcus* sp.**	-	-	5
	***Staphylococcus warnerii***	1	2	1
	***Streptococcus gordonii***	-	-	2
	***Streptococcus mitis***	4	6	3
	***Streptococcus mutans***	-	4	1
	***Streptococcus oralis***	-	-	1
	***Streptococcus parasanguis***	-	2	-
	***Streptococcus pneumonia***	-	2	-
	***Streptococcus salivarius***	-	3	-
	***Streptococccus sanguinis***	-	3	2
	***Streptococcus sanguis***	-	1	-
	***Veillonella dispar***	-	2	-
***Sub-total***		**6**	**42**	**26**
**Sub-total**		**11**	**83**	**55**
**Total**		**149**		

160 strains isolated in total: 149 strains in total identified to different levels; 11 strains were not amenable to identification, of which 7 strains were lost during sub-culture, the DNA of 2 strains could not be amplified by PCR and the amplicons of 2 strains could not be sequenced. *Sequence matched a database phylotype

The figures in boldface are subtotals

The bacterial group origin by cavity and restoration are presented in Table 4. The most frequently isolated genera by sample origin were *Staphylococcus* and *Streptococcus* (from 5/9 teeth), and *Actinomyces* and *Lactobacillus* (from 4/9 teeth). Of these four groups, the most frequently isolated species were *Staphylococcus warneri*, *Streptococcus mitis*, *Actinomyces naeslundii*, and *Lactobacillus casei*.

Other commonly isolated Gram-positive species included: *Rothia dentocariosa* (2/9 cases from both cavity and amalgam); *Propionibacterium acnes* (2/9 cases from both cavity and amalgam); and *Gemella morbillorum* (from cavity wall in a single specimen). The Gram-negative species isolated were *Capnocytophaga haemolytica*, *Haemophilus parainfluenzae*, *Neisseria sicca*, and *Veillonella dispar*, each isolated from the cavity wall of one tooth, whilst *Neisseria pharyngis* was isolated from the amalgam surface of another tooth.

## Discussion

This study examined the nature and extent of bacterial colonisation on both restoration and cavity wall surfaces after long-term clinical service of mostly amalgam restorations using two different outcome measures (SEM observation and bacterial culture). It was also a validation of a shorter-term in vitro study evaluating microbial leakage around amalgam restorations [20]. Clinically, the restoration margins appeared “serviceable”, that is, they were not deemed to require immediate replacement, but the tooth required extraction for other reasons, usually periodontal disease.

Twenty teeth with amalgam or GIC restorations surviving in the mouth for 2–20 years were observed under SEM after extraction and processing. SEM allows the entire exposed cavity wall/restoration interface to be visualised and evaluated at low through to high magnification [23]. Potential artefacts of SEM processing, including distortion and translocation, may be relatively easily detected. Although bacterial biofilms in the cavity/restoration interface may theoretically be disturbed during the tooth splitting process, in most cases, there was clear evidence of separate biofilms often with different surface morphological characteristics on the apposing walls. A “single biofilm mass” filling the cavity-restoration interface could theoretically be divided during the splitting process but would then be expected to exhibit complementary surface characteristics rather than the distinctly different surfaces evident. Transmission electron microscopy was not deployed because of difficulties in sectioning through materials of different properties (dentine, amalgam), coupled with the fact that only relatively small areas could be viewed at any one time, requiring correlative light microscope evaluation for context.

The need to split the tooth to yield halves with and without the restoration led to the loss of 7 of the original 27 teeth. The method was suitable for the intended examination in the majority of cases, although the mode of splitting may arguably favour teeth with a restoration gap likely to encourage microbial in-growth, thus the 7 excluded teeth may potentially have exhibited less leakage, although this was not verifiable.

There was a positive association between the presence of bacteria on the cavity wall and the restoration surface in just over half the cases, 11/20 specimens displaying presence on corresponding surfaces. The SEM study showed no intuitively obvious relationship between number of restoration surfaces, their duration of service, and presence of microorganisms, the sample size being too small for formal statistical evaluation.

Although many teeth displayed a thick external surface biofilm, its down-growth into the cavity/restoration interface was not consistently observed, even when there were apparent gaps (Fig. 4 (1)). It is possible that in the intact tooth/restoration complex, separate gaps may not coalesce to form a continuous path, precluding colonisation of isolated or sequestered gaps. Such bacteria-free voids were also observed at the exposed interface in an 8-week experimental study [20].

In general, where bacteria were present, a gradual reduction in obvious biofilm was evident as the interface was traced from the edge of the cavity to its base. Several specimens revealed an irregular layer whose appearance varied from a lobulated to a matted surface hinting at bacterial presence underneath. Bacterial biofilms contain extracellular polysaccharide matrix and, although this component may be reduced during sample processing for SEM, its presence may obscure visibility of the underlying cellular morphotypes [23]. Another explanation for obscured visibility of biofilms may be their mineralisation, possibly explaining the absence of evidence for bacteria either on the tooth or restoration in teeth filled at least 2–5 years previously. Calculus formation is a common oral phenomenon; one of the theories of its formation is calcium precipitation and phosphate transport by the bacteria [24]. *Corynebacterium matruchotii* is associated with dental calculus formation and has been shown to produce a membrane-associated proteo-lipid capable of inducing hydroxyapatite formation in vitro [24]. This particular strain has been isolated from the cavity/restoration interface. The possibility that the observation represented a smear layer [25] was precluded by the thickness of the observed layer (30 μm) covering large areas, whereas smear layers are 1–4 μm thick covering only instrumented surfaces [26]. In addition, it is unlikely that smear layers would survive for the durations for which the restorations were in clinical service. The possibility that the observed structure represented Ca(OH)_2_ lining is excluded by its potential to dissolve and leak out over relatively shorter periods [27]. The possibility of the layer representing amalgam corrosion products may be ruled out by the fact that they resemble crystals more than a lobulated surface [28]. The growth and survival of bacteria in this micro-niche may be influenced by the interaction of several factors, including nutrient gradients, redox potential, chemical composition of surfaces, and pH [29].

The few GIC restorations (3/20) all exhibited extensive and varied bacterial biofilms on the surfaces, many microns thick, in places. GIC may become rough and porous (Fig. 3 (1)) when exposed to the oral cavity [30], and when placed in bulk may contract on curing, allowing breakage of adhesive bonds from one of the apposing surfaces, allowing bacterial ingress.

The bacterial decontamination, sampling, culture, and identification protocols adopted are well-established [21, 31–33]. The negative sterility controls proved the effectiveness of the decontamination procedures. Positive cultures were obtained from all test tooth samples, either from the cavity or amalgam surfaces but not from all surfaces; 3 cavity and 1 restoration surfaces yielded no isolates (Table 4). The findings appear to corroborate the SEM observations (absence of visible bacteria on cavity wall and corresponding amalgam surface of 7 teeth), although the culture absence may also be explained by a viable but non-culturable state. That, corresponding surfaces of cavity and restoration did not always share bacterial types (Table 4), adds to the case for separate biofilms on apposing surfaces. It is speculated that the restoration-cavity interface may harbour a single restricted niche when the marginal gap is small but potentially multiple variable niches, when the gap is larger. The larger gap allows different environments to develop and therefore differing microbial communities.

Nine teeth exhibited an ‘irregular amorphous layer’ on the cavity wall and/or amalgam. If this layer were in fact a mineralised bacterial biofilm, paper points would fail to sample it, whereas a steel bur may prove effective. The frequency of colony-forming units grown from the different sampling approaches tends to reinforce this theory. Most isolates in the current study were recovered by scraping a bur across the amalgam or dentine surface (47%); the rest were sampled by gently drilling with the bur (34%) or using paper points (19%). It must be added that there was no clinically obvious softening of the dentine consistent with active caries, and this is supported by the higher CFU yield from a hand-held bur rather than a rotating bur. This finding is not inconsistent with observations of taxa sampled from the canal wall, compared to dentine structure [34, 35].

Statistical correlations could not be made between the clinical and culture data due to the relatively small sample size, and indeed, the design was guided by the desire to produce descriptive data at this stage. Nevertheless, the descriptive data are suggestive of a negative association between the number of species isolated and the longevity of the restoration. Amalgam restorations less than 5 years old yielded nearly twice the number of isolates compared to those present for more than 5 years, a trend similar to the SEM observations. Microleakage around amalgam restorations putatively decreases with time [36–38] due to obstruction of the leakage pathways by corrosion products. Another possible explanation advanced in this study is mineralisation of the bacterial biofilm or formation of calculus in the cavity restoration interface over time.

The age of restorations in the culture study ranged between 0.6 and 20 years. The carious lesions in 5/9 teeth were confined to a single surface (Table 2), allowing cavity and restoration sampling from a caries-free area. Most isolates identified were facultative (92%) with a relatively low proportion of obligate anaerobes (8%). The most commonly isolated genera, *Staphylococcus*, *Streptococcus*, *Actinomyces*, and *Lactobacillus*, are associated with root caries [39, 40] and root-treated teeth with persistent apical periodontitis and coronal leakage [41].

Oral microbial biofilm models [20, 42, 43] and in situ caries risk studies [44] give some insight about early colonisation and microbiota behaviour, but they do not predict longer-term behaviour. Such studies have helped to elucidate the critical threshold of marginal gap size required to facilitate or impede caries lesions, but with contradictory findings [45, 46]. Clinical evidence suggests that even the “best-fit” apposing surfaces, such as those arising from incipient cracks in teeth maybe colonised by bacteria [47, 48].

The origin of microbiota recovered from marginal gaps may be the oral environment [8], residual dentinal infection [35], or residual caries [49]. Once established in a mature biofilm, they may ultimately contribute to pulpal inflammation, root canal infection, and subsequent periapical inflammation. Despite its putative importance as the precursor of endodontic problems, the tooth/restoration interface microbiota has been characterised only to a limited extent.

In vivo bacterial culture and histological techniques have brought some insight about the relationship between bacterial presence under restorations and pulpal inflammation [50–53]. Cultivable microbiota grown over a period of 4–6 weeks under composite restorations resemble aged dental plaque [50] rather than that typically associated with carious lesions [8]. An equal proportion of facultative and anaerobic bacteria have been reported, mostly Gram-positive, with streptococci, peptostreptococci, and *Actinomyces* predominating, and lactobacilli absent [50]. Microbial leakage around a variety of restorative materials placed in a monkey model [8], measured at 2–3- and 8-week post-placement, using anaerobic techniques revealed Gram-negative and Gram-positive anaerobic rods and cocci (including *Bacteroides* and *Fusobacterium* species)*.*

Marginal adaptation has been shown to influence the invading microbiota. Varpio et al. [54] studying composite restorations in service for 3 years in extracted primary molars found that clinically “excellent” restorations were bacteria-free in 25% of cases but the rest exhibited bacteria under fillings (75%) and in dentinal tubules (61%). They also found that marginal discolouration was associated with marginal defects, bacterial leakage, and pulpal reactions (necrotic in 7/16). Cavities with marginal restoration ditching greater than 0.4 mm harboured significantly more cultivable bacteria (mutans streptococci and lactobacilli) than those narrower than 0.4 mm [55]. The phenotypic and genotypic diversity of surviving cultivable microbiota beneath well-sealed restorations was less complex and dominated by bacteria producing glycosidic enzymes capable of releasing sugars from glycoproteins [49, 56]. The latter found significantly less bacteria when well-sealed restorations were placed after incomplete caries removal compared to complete caries removal.

Splieth et al. [57] found that restoration type, caries activity and its location influenced restoration-cavity interface microbiota. In contrast, Mo et al. [58] found no effect of material type or caries location on microbiota, which was dominated by anaerobic species; however, they found the diversity was significantly different between experimental groups classified by material and cavity type.

There is little insight about the relationship between clinical marginal restoration/cavity gap size and longer-term microbial leakage dynamics in apparently well-placed restorations and longer-term pulp breakdown [11, 59].

The main species associated with carious dentine are *Streptococcus* spp., *Actinomyces* spp., *Lactobacillus* spp., and other Gram-positive rods [60]. Although a specific role was proposed for *Actinomyces* as the main causative factor in root caries [61], the presence of a site-specific niche was challenged [62]. Initial colonisers such as *Streptococcus* spp. (commonly *Streptococcus mitis*) and *Actinomyces* spp. (commonly *Actinomyces naeslundii*) have been shown to be predominant in root and crown caries. Furthermore, *Corynebacterium*, *Rothia*, *Propionibacterium*, *Veillonella*, *Neisseria*, *Gemella*, and *Haemophilus* species have also been isolated from ‘active’ carious lesions [39]. These findings correlate with the species identified in this study, potentially inviting the suggestion of miss-sampling from adjacent caries in some teeth, but this was categorically not the case for reasons advanced above. Furthermore, the presence of caries, elsewhere in the tooth, did not predict bacterial presence in the interface.

Gram-positive obligate anaerobes (*Propionibacterium acnes*, *Eubacterium* spp*.*, *Propionibacterium propionicus*, and *Bifidobacterium* spp.) have been found in deep carious dentine [34] by sampling from progressively deeper dentine layers. Of the cultivable bacteria in the current study, only 8% were obligate anaerobes, perhaps because only the surface of the dentine and restoration were sampled, resulting in a higher proportion of facultative anaerobes. Of the 160 isolates recovered in this study, 11 remained unidentified, of which 7 were lost during the process of sub-culturing to purity. It is likely that these were fastidious organisms reliant on co-colonisers or stored nutrients for sustenance [35, 63, 64]. Two isolates did not yield PCR amplicons, and a further two yielded PCR amplicons that were not amenable to yielding useable sequences for reasons that were unclear.

Enamel caries may involve a specific set of bacterial species because of the requirement of initial sustained demineralisation to breach a principally mineralised tissue. Root caries, on the other hand, may involve different microbiota because invasion of cementum and dentine provide different conditions [65]. In this context, the cavity/restoration interface may be considered already “breached” and mimic the latter condition, potentially explaining the low recovery of *Streptococcus mutans*. The less acidogenic bacteria, such as non-mutans streptococci (*Streptococcus sanguinis*, *Streptococcus mitis*) may reduce the pH to a level sufficient to cause dentine demineralisation. The attempts to classify root caries microbiota have been fraught with sampling technique problems, as it is easily contaminated by surface plaque [40]. The latter, comparing sound, leathery and soft root carious dentine, found that whilst *Streptococcus mutans* was a constant presence, *Lactobacillus* spp., *Actinomyces* spp., and non-mutans streptococci progressively increased. The frequent occurrence of these latter species in the current study may suggest that the cavity-restoration microbiota is similar to that of ‘soft root caries’.

An improved sampling method, avoiding contamination from the superficial plaque, isolated *Staphylococcus epidermidis* and *Staphylococcus haemolyticus* with greater frequency from active and arrested carious lesions compared to sound root surfaces [39]. The presence of staphylococci has often been deemed to infer contamination of the sample; however, their presence has been confirmed in oral samples and traced to the patient’s own staphylococcal strains of nasal origin [66] and possibly food sources [67]. Furthermore, many cultural and molecular studies of tooth root canals [41, 68] and tooth caries [39] have confirmed their presence. Despite the fact that coagulase-negative *Staphylococcus* species may be confused with the *Streptococcus mitis* group when using 16S rRNA sequences, it is still believed that staphylococci are legitimate colonisers and not merely contaminants; the discrimination between the mitis-group streptococci and staphylococci may be improved by comparison of the *SodA* gene [69]. The high frequency of *Staphylococcus* spp. in the present study may possibly reflect secondary caries transitioning between states of activity and arrest.

The cultivable microbiota in root canals of root-treated teeth associated with persistent apical periodontitis and coronal leakage [41] was also found to be similar to the bacterial profile in this study. The implication is that the microbiota described in this study is characteristic of coronal leakage that may facilitate the dynamic transition between dentine softening and caries arrest, whilst also seeding root canal system infection. These notions of microbiotal shifts are consistent with the current popularity of the dysbiosis theories in explaining transitions from health to disease [70, 71].

## Conclusions

This study provides observational insight into the morphological distribution of microbiota in tooth/restoration interfaces exposed to the oral environment for periods up to 20 years. Gaps were not always colonised, but when they were, there was a higher chance that both apposing surfaces would be. Biofilms on apposing surfaces, although sharing taxa, appeared to be unique and separate. Bacterial penetration from the marginal opening into the cavity-restoration interface varied but was qualitatively similar to that of soft root caries and pulp spaces of root-treated teeth with apical periodontitis and coronal leakage. Apparently, mineralised material (calculus-like) filling the cavity/restoration interface may explain the longevity of restorations despite the presence of obvious gaps.
